# Identification of Immune Traits Correlated with Dairy Cow Health, Reproduction and Productivity

**DOI:** 10.1371/journal.pone.0065766

**Published:** 2013-06-12

**Authors:** Georgios Banos, Eileen Wall, Michael P. Coffey, Ainsley Bagnall, Sandra Gillespie, George C. Russell, Tom N. McNeilly

**Affiliations:** 1 SRUC, Roslin Institute Building, Edinburgh, United Kingdom; 2 Faculty of Veterinary Medicine, Aristotle University of Thessaloniki, Thessaloniki, Greece; 3 Moredun Research Institute, Edinburgh, United Kingdom; INRA, France

## Abstract

Detailed biological analyses (e.g. epidemiological, genetic) of animal health and fitness in the field are limited by the lack of large-scale recording of individual animals. An alternative approach is to identify immune traits that are associated with these important functions and can be subsequently used in more detailed studies. We have used an experimental dairy herd with uniquely dense phenotypic data to identify a range of potentially useful immune traits correlated with enhanced (or depressed) health and fitness. Blood samples from 248 dairy cows were collected at two-monthly intervals over a 10-month period and analysed for a number of immune traits, including levels of serum proteins associated with the innate immune response and circulating leukocyte populations. Immune measures were matched to individual cow records related to productivity, fertility and disease. Correlations between traits were calculated using bivariate analyses based on animal repeatability and random regression models with a Bonferroni correction to account for multiple testing. A number of significant correlations were found between immune traits and other recorded traits including: CD4^+^:CD8^+^ T lymphocyte ratio and subclinical mastitis; % CD8^+^ lymphocytes and fertility; % CD335^+^ natural killer cells and lameness episodes; and serum haptoglobin levels and clinical mastitis. Importantly these traits were not associated with reduced productivity and, in the case of cellular immune traits, were highly repeatable. Moreover these immune traits displayed significant between-animal variation suggesting that they may be altered by genetic selection. This study represents the largest simultaneous analysis of multiple immune traits in dairy cattle to-date and demonstrates that a number of immune traits are associated with health events. These traits represent useful selection markers for future programmes aimed at improving animal health and fitness.

## Introduction

The difficulties in systematic recording of complex health and fitness traits present formidable limitations to detailed biological analyses. For example, temporal and spatial epidemiological studies necessitate close monitoring of large populations of animals. Further, modern breeding programmes require field data on important functions associated with animal health, fitness and welfare [1].

The lack of large-scale recording of such traits calls for the development of alternative measures. One approach involves the identification of easily measurable immune traits which may predict an individual’s susceptibility to disease or give a measure of an individual’s general immune-competence. When available in large-scale, this type of data may be used in studies aiming to better understand the background of a disease. In addition, such proxy markers would provide a basis for predictive models of animal health and fitness when combined with routinely recorded data (e.g. fertility and production). However, to utilise immunological traits properly it is necessary to first define their associations with health and fitness traits within animal populations.

The concept of using immune traits as markers for the identification of individual livestock with improved health has primarily been explored in pigs and dairy cattle, where studies have largely focussed on estimating heritabilities of immune traits in healthy individuals [2]–[5] or calculating estimated breeding values for selected immune traits [6], rather than exploring actual associations between immune traits and health. Where these associations have been studied, associations have generally been identified at the population level, comparing mean levels of immune measures in groups of animals with either high or low susceptibility to disease. Examples of this include comparisons of innate immune traits such as acute phase protein levels and innate immune cell function in pig breeds with differing generalised susceptibility to disease [7], analysis of lymphocyte subpopulations in groups of mastitis-resistance or susceptible cows [8], comparison of natural antibody levels in groups of cows with or without a history of clinical mastitis [9], comparison of acute phase protein levels in cattle breeds with differing susceptibility to theileriosis [10] or comparison of disease incidence in groups of cows with high, average or low estimated breeding values for antibody and/or cell-mediated immune responses [11]. However, as these studies are mainly based on comparisons at group level of each immune parameter, they may have only limited predictive capacity in determining a particular individual’s susceptibility to disease. In order to identify and properly quantify associations between immune traits and disease resistance, detailed information on disease phenotypes at the individual animal level is required. Furthermore, combining these analyses with detailed production and performance data may allow any trade-offs between disease resistance and animal productivity to be identified.

One population with the required level of phenotypic data is the Crichton Dairy Research herd in Scotland housing the Langhill lines of dairy cows, a population of Holstein dairy cows which has been used for genetic and nutritional research into dairy cow health, welfare and productivity for over 30 years [12], [13]. The herd is comprised of two genetic groups, one selected for the highest levels of milk protein and fat and the other producing milk with the UK national average of protein and fat. These genetic groups are additionally assigned to two distinct nutritional regimes, a high forage, low concentrate diet and a low forage, high concentrate diet more typical of UK dairy cow herds. Both diet and genetic group has previously been shown to influence dairy cow health with cows in the genetically select group or those fed the high concentrate diet having more health problems [12], [13].

The objective of this study was to analyse a number of cellular and serological immunological parameters within this herd at multiple time points in order to (i) estimate correlations between immune traits from blood sample analysis and individual health and performance traits, (ii) assess the between-animal repeatability of each immune trait and (iii) examine the influence of genetic line and diet group on immune traits. The underlying aim was to study immune traits in relation to health events both at the individual and at the group level. The immunological traits were selected with no focus on specific pathogens to provide measures of the underlying immunological fitness of each animal.

Selected immune traits included serum levels of the pro-inflammatory cytokine TNFα, the expression of which has been associated with disease susceptibility and inflammatory conditions [14], the acute phase protein haptoglobin which is involved in a number of inflammatory conditions of cattle [15], [16] and has been previously associated with breed specific disease resistance in cattle [10], and natural antibodies (NAb) which are thought to be an important antibody component of the innate immune response and appear to be related to frequency of mastitis [9]. In addition to these serological traits, the proportions of various blood leukocyte subsets including innate immune cells such as monocytes, natural killer cells, neutrophils and eosinophils, and adaptive immune cells including T and B lymphocyte subsets were also determined.

The hypothesis addressed in this study was that the levels of specific cellular or serological measures of immune system status could be correlated with health, fitness and production traits within this herd, and that certain immune measures exhibit between-animal variation and repeatability and could therefore potentially be used to help understand dairy cow health in the field.

## Methods

### Animals

Data were from 248 Holstein cows raised in the SRUC Crichton Dairy Research herd in south-west Scotland, which houses the Langhill lines of dairy cattle. Cows were born between 16 January 2003 and 21 March 2009 and at the time of this study were between their 1^st^ and 6^th^ lactation inclusive. Cows were participating in an ongoing genetic selection experiment and were evenly distributed between two different genetic groups (control and select). Briefly, control group cows were daughters of sires with average genetic merit for milk fat plus protein yield on the UK scale, whereas select group cows were daughters of sires with the highest genetic merit for kg fat plus protein chosen at the time of artificial insemination. Within each genetic group, animals were randomly allocated to either a high concentrate (low forage) or a low concentrate (high forage) diet group at first calving, as part of an ongoing feed experiment [13]. The high concentrate diet is representative of intensively raised dairy cattle in high-input commercial systems whereas the low concentrate group mimics conditions in low-input grazing systems. Animal allocation to genetic and diet groups was performed in such a way as to keep the groups balanced for number of cows and sires.

### Ethics Statement

Blood sample collection was conducted in accordance with UK Home Office regulations (PPL No: 60/4278 Dairy Systems, Environment and Nutrition) and procedures were approved by the SRUC Animal Experimentation Committee. Otherwise, the study was restricted to routine on-farm observations and measurements that did not inconvenience or stress the animals.

### Immune Traits

Blood samples were collected on five separate occasions at two-monthly intervals between July 2010 and March 2011. An average of 177 serum samples were analysed at each time-point (range = 172–187) and represented all lactating dairy cows within the herd at the time of sampling. An average of three serum samples per cow were analysed over the study period (range = 1–5 serum analyses per cow). Flow cytometric analysis was performed on 48 samples per time-point due to logistical constraints and included only cows within the high concentrate diet group which, as explained, reflects the practices in conventional high-input systems. The relatively small number of samples which could be analysed at each time-point did not allow the low concentrate diet group to be included as this would have led to very few cows per line-group combination. However, both genetic lines were equally represented in this subset. An average of three samples per cow were analysed by flow cytometry over the study period (range = 1–5 flow cytometric analyses per cow).

For serological analyses, whole blood was collected into plain Vacutainers (BD) and blood allowed to coagulate before centrifugation at 12,000 × *g* for 10 min. The concentration of serum haptoglobin was measured using a Tridelta PHASE™ Haptoglobin Assay kit (Tridelta Development Ltd, Maynooth, IR) according to the manufacturer’s instructions and expressed as µg/ml. Serum Tumour Necrosis Factor Alpha (TNF-α) concentrations were determined by ELISA using a Bovine TNF-α DuoSet ELISA kit (R&D Systems, Inc., Minneapolis, MN) according to the manufacturer’s instructions, and expressed as pg/ml. Natural antibody (NAb) levels were measured by ELISA specific for an antigen unlikely to be specifically recognised by cattle serum antibodies as follows: microtitre plates (Immunolon 2 HB, Thermo Electron Corporation, Milford, MA) were coated overnight at 4°C with 1 µg/ml endotoxin-free Keyhole Limpet Hemocyanin (KLH) (Calbiochem®, Nottingham, UK) in 0.1 M carbonate buffer pH 9.6. After washing in PBS, pH 7.4, containing 0.05% Tween 20® (PBS/T), non-specific binding sites were blocked with PBS containing 3% fish gelatin (Sigma-Aldrich, St. Louis, MO) for 1 hr at 37°C. Plates were subsequently incubated with serum diluted 1∶40 in PBS containing 0.5 M NaCl and 0.5% Tween 80® (PBS/NaCl/T) for 1 hr at 37°C. For each plate, 8 wells were incubated with PBS/NaCl/T alone (blank) and a known positive control sample was analysed. Each sample was analysed in duplicate. After washing in PBS/T, plates were incubated for 1 hr at 37°C with sheep anti-bovine IgG (H+L chain) conjugated to horseradish peroxidase (AbD Serotec, Kidlington, UK) diluted in PBS/NaCl/T. After a final wash in PBS/T, colour reactions were developed by addition of Sigma-Fast OPD substrate (Sigma-Aldrich). Reactions were terminated after 5–10 min by addition of 2.5 M H_2_SO_4_, and the optical density (OD) at 492 nm (OD@492) measured using a Sunrise™ microplate reader (Tecan, Männedorf, CH). The dilution of serum used for this assay was chosen following serial dilution of representative samples to ensure that the OD@492 for all samples was on the linear part of the curve. For each plate, the mean OD@492 of the blank wells was subtracted from the sample readings, and inter-plate variation was normalised to the positive control sample.

For flow cytometric analysis of circulating leukocyte populations, blood samples were collected into Lithium heparin Vacutainers (BD, Franklyn, NJ) and red blood cells lysed by addition of 5 ml of warm ammonium chloride lysis solution (1.5 M NH_4_Cl, 100 mM NaHCO_3_, 10 mM Na_2_EDTA) to 1 ml heparinised blood with gentle mixing. Leukocytes were pelleted by centrifugation at 850 × *g* for 10 min, washed once in 8 ml PBS and re-suspended in 1 ml PBS. Fifty µl of leukocyte suspensions were transferred to a round-bottomed 96 well plate, washed once in FACS buffer (5% foetal calf serum, 0.05% sodium azide in PBS) and incubated for 30 min at 4°C in FACS buffer containing mouse monoclonal antibodies (mAbs) against bovine CD3, CD4, CD8, CD14, CD21, CD335, γδ TCR, or appropriate isotype control mAbs. Details of mAbs and their cellular targets are shown in Table 1. CD4/CD8 labelling was performed simultaneously to provide accurate estimates of CD4:CD8 T cell ratios. After mAb incubation, cells were washed twice with FACS buffer. For indirect labelling (CD3 and pan γδ TCR) cells were subsequently incubated with goat anti-mouse IgG [H+L] conjugated to Alexa Fluor® 647 (Invitrogen, Carlsbad, CA) for 30 min at 4°C. After final washing, cells were fixed in 1% PFA in PBS for 10 min at RT before analysis on a BD FACSArray™ Bioanalyser (BD Biosciences, San Jose, CA) using the manufacturer’s acquisition software. Data analyses were performed using FlowJo version 7.6.1 analysis software (TreeStar, San Carlos, CA). The results were expressed as the percentage of cells within the peripheral blood mononuclear cell (PBMC) population which were positive for each surface marker. In addition, differential cell counts were performed by analysis of unstained cells and identifying leukocyte populations by their size (forward scatter), granularity (side scatter), and auto-fluorescence as previously described [17]–[19]. Specifically, the proportions of different leukocyte subsets (lymphocytes, monocytes, neutrophils and eosinophils) were assessed based on morphological features (size, granularity) and autofluorescence (for eosinophils); while the proportions of specific subsets of peripheral blood mononuclear cells (T and B lymphocytes, monocytes and CD335^+^ natural killer cells) were assayed by staining with subset-specific monoclonal antibodies (Table 1). Total leukocyte counts were performed using a Neubauer haemocytometer.

**Table 1:** Details of mouse monoclonal antibodies (mAb) used for flow cytometry.

mAb	Specificity	Isotype	Conjugation	Cellular expression	Source
CC8	CD4	IgG2a	Alexa Fluor® 647	Helper T cells [64]	AbD Serotec^1^
CC63	CD8	IgG2a	R-phycoerythrin	Cytotoxic T cells [65]	AbD Serotec^1^
TÜK4	CD14	IgG2a	Alexa Fluor® 647	Monocytes, macrophages [66]	AbD Serotec^1^
CC21	CD21	IgG1	R-phycoerythrin	B cells [67]	AbD Serotec^1^
AKS1	CD335 (NKp40)	IgG1	R-phycoerythrin	Natural killer cells [39]	AbD Serotec^1^
MM1A	CD3	IgG1	n/a	Pan-T cells [68]	VMRD Inc.^2^
GB21A	γδ T cell receptor	IgG2b	n/a	Pan-γδ T cells [69]	VMRD Inc.^2^
P3.6.2.8.1	Isotype control	IgG1	R-phycoerythrin	n/a	eBioscience^3^
eBM2a	Isotype control	IgG2a	R-phycoerythrin	n/a	eBioscience^3^
eBM2a	Isotype control	IgG2a	Alexa Fluor® 647	n/a	eBioscience^3^
VPM21	Isotype control	IgG1	n/a	n/a	MRI^4^
VPM22	Isotype control	IgG2b	n/a	n/a	MRI^4^

1 AbD Serotec, Kidlington, UK;

2 VMRD Inc., Pullman, WA;

3 eBioscience, San Diego, CA;

4 Moredun Research Institute; n/a = not applicable.

Intra-assay repeatability for flow cytometry assays and cell counts was measured by performing five replicates of the assays on blood samples from three healthy donor cattle. Inter-assay precision was measured by repeating the flow cytometry assays and cell counts on three separate blood samples collected from same three donor animals on the same day. Intra-assay repeatability and inter-assay precision estimates were expressed as % coefficient of variation (CV%).

### Health Event Traits

All cows in the Crichton Dairy Research herd were subject to routine technician and veterinary monitoring and all treatments made were entered into the research database. Codes referring to different treatments have been established as routine practice. For the purposes of the present study, these codes were extracted from the database and grouped into three main trait categories: (i) clinical mastitis, (ii) reproductive problems (including mucopurulant exudates, metritis, pyometra, whites, cystic ovaries, retained placenta, abortion, dull post calving and down post calving), and (iii) lameness (including sole ulcer, digital dermatitis, interdigital granuloma, thin sole, white line disease, slurry heel, bruising and swollen hock etc.). In addition to health and disease, many of the recorded traits also have animal welfare implications.

Each cow was first scored as 0 or 1 on the day of the immunological analysis for each of the three health categories, where 0 means absence and 1 presence of treatment within a week before or after that day. Subsequently, the total number of days of treatment for each category was calculated for the lactation and was converted to the number of distinct episodes per lactation. Following veterinary consultation, distinct episodes were assumed for consecutive treatments more than 7, 21 and 28 days apart for clinical mastitis, reproductive problems and lameness, respectively. A similar definition for clinical mastitis is used in the UK national evaluation system [20]. However, alternative definitions of distinct episodes were also considered by changing these durations by as much as 20% (e.g. 22 to 34 days for lameness) in order to test the sensitivity of our results.

### Reproductive Performance Traits

A total of 8 reproduction traits were derived from the official farm records. These traits are listed in Table S4 and pertained to the lactation during which the cow was sampled for immunological analyses. Thus, calving interval referred to the interval between the calving preceding this lactation and this calving. Number of services referred to number of artificial inseminations required for conception. Calving performance was expressed as a dystocia score of 0 for normal calvings and 1 otherwise. Stillbirth was expressed as 0 for calves born alive and 1 for those born dead or died within 24 hours. Both dystocia and stillbirth referred to the calving preceding the current lactation. In addition to reproduction, both these traits are also important from an animal welfare perspective.

### Lactation Traits

These traits pertained to milk production, health, feed consumption, body energy and feed efficiency measured throughout a cow’s lactation. Data included daily milk yield (sum of 3 milkings), daily live weight (average of 3 daily weighings) and weekly body condition score, weekly milk fat and protein yield and milk somatic cell count, and thrice weekly feed and dry matter intake. Weekly averages for these traits were calculated. Milk somatic cell count was of interest as an indicator of (sub)clinical mastitis [21]. Additional traits included empty body weight (live weight adjusted for the weight of feed and the foetus) and the ratios of feed intake to milk yield and dry matter intake to milk yield; the latter were indicators of the efficiency with which feed was converted to milk. A total of 11 lactation traits were defined and matched to the cows’ immune profile. Weekly records of these traits were kept across the entire standard 305-d (www.icar.org) lactation during which the immunological analysis took place.

### Statistical Analysis

The statistical analysis of immune traits was conducted with a repeatability model that included the effects of genetic line, diet group (except for the flow-cytometry traits), year by month of calving interaction, lactation number by age at calving interaction, and week of lactation when the measurement was taken. The latter effect was intended to account for possible systematic variation in metabolic and hormonal conditions associated with the different stages of lactation. Cow was also fitted as a random effect to account for variation between individual animals. Based on this model, the impact of genetic and diet groups was calculated adjusted for all other sources of systematic variation. The model also yielded estimates of between-animal repeatability for each immune trait.

Correlations between individual immune traits were estimated in a series of bivariate analyses using the same repeatability model. Both total phenotypic as well as animal correlations were calculated. The latter represents the association between traits that is ascribed to specific attributes of the animals including their genetic profile and heritable factors. Total phenotypic correlations then comprised correlations due to the animal effect and correlations due to residual, unexplained sources of variation (e.g. the environment).

Another series of bivariate analyses based on the same model yielded correlations between immune traits and health events. Firstly, records of the 16 immune traits were analysed jointly with the 3 health event traits expressed as 0 or 1 on the week of the immunological measurement. The correlation between the immune traits and number of episodes for each health event trait was estimated with a single observation model that excluded the random cow term. This was done because only a single record of number of episodes for the lactation in question was available per cow.

A similar set of repeatability model analyses were conducted between the 16 immune traits and 11 lactation traits that corresponded to the week of the immunological measurement. Correlation estimates described the association of immune traits with lactation traits measured on the same week.

In a separate set of bivariate analyses, total phenotypic and animal correlations between immune traits and weekly lactation traits across the entire lactation were estimated. Here, immune traits were analysed with the same repeatability model as before but lactation traits were analysed with a random regression model which included third order polynomials for both the fixed curve and the random individual animal deviations from the curve. This model allowed the estimation of covariances between successive weekly records of the same animal accounting for the impact one such record has on the following record.

Finally, correlations between the 16 immune traits and the 8 reproductive performance traits were estimated with a single observation model that excluded the random cow term and yielded only phenotypic correlation estimates. This was done because only a single record of reproductive performance in the lactation in question was available per cow.

The basic models of statistical analysis described above are also presented in mathematical form in Appendix S1.

Because multiple testing was performed in the analyses described above, some of the significant results might be attributed to randomness. For this reason, a strict Bonferroni correction was implemented [22].

All statistical analyses were conducted using the ASREML software package [23]. Binary traits (dystocia, stillbirth rate, clinical mastitis, reproductive problems and lameness) were analysed using a logit function. Count traits (number of clinical mastitis, reproductive or lameness episodes, and number of inseminations) were analysed using a Poisson function. Milk somatic cell count records were log-transformed prior to the analyses to ensure normality. *P* values <0.05 were considered significant.

## Results

### Intra-assay Repeatability and Inter-assay Precision for Cellular Immune Trait Analyses

Intra-assay repeatability and inter-assay precision estimates for flow cytometry and cell count measurements are summarised in Table 2. For flow cytometry assays, the results of which were expressed as the % of each cell type within the PBMC or total leukocyte population, CV% values were ≤9.4 for intra-assay repeatability and ≤9.0 for inter-assay precision. For cell counts CV% values were between 14.4–96.2% for intra-assay repeatability and 30.0–47.9% and intra-assay precision. Due to the poor repeatability and precision of cell count measurements, all subsequent analyses of cellular immune traits were performed using leukocyte subset measurements expressed as a proportion of the total PBMC or leukocyte population, rather than as a count/ml blood.

**Table 2:** Estimated intra-assay repeatability and inter-assay precision for cellular immune trait measurements.

Cellular immune traitmeasurement	Intra-assay repeatability (CV^1^%)	Inter-assay precision(CV^1^%)
Total leukocyte count	14.4–96.2	30.0–47.9
% PBMC^2^	0.3–0.6	0.6–1.5
% CD3^+3^	0.3–1.6	0.2–0.4
% CD4^+3^	1.7–7.0	2.9–3.0
% CD8^+3^	1.6–5.2	2.1–4.8
% CD14^+3^	1.9–4.5	1.4–5.1
% CD21^+3^	1.6–4.8	3.0–8.1
% CD335^+3^	5.3–9.4	5.7–9.0
% γδ TCR^+3^	0.3–2.0	0.3–1.3
CD4^+^ : CD8^+^ ratio	1.7–7.0	2.9–3.0
% Lymphocytes^4^	0.3–0.9	0.5–2.1
% Monocytes^4^	1.6–4.3	1.6–3.4
% Neutrophils^4^	2.7–7.2	1.9–5.9
% Eosinophils^4^	2.0–6.9	2.0–6.7

1 Coefficient of Variation;

2 Peripheral Blood Mononuclear Cells;

3 % of PBMC that were CD3, CD4, CD8, CD14, CD21, CD335 and γδ TCR positive;

4 % of total leukocytes that were lymphocytes, monocytes, neutrophils or eosinophils.

### Descriptive Statistics, Variability and Repeatability Estimates of Recorded Traits

The datasets for the analyses of immune, lactation and reproductive performance traits are summarised in Tables 3–5, respectively. Coefficients of variation were calculated to determine the variability of each trait. These ranged from 23 to 221% for immune traits, 14 to 315% for lactation traits and 17 to 118% for reproductive performance traits (Tables 3, 4, and 5, respectively) indicating considerable differences in variability between the different traits recorded. Tables 3 and 4 also show estimates of immune and lactation trait variation that is due to differences among individual animals (the animal variance), for the traits with repeated records. The proportion of the total phenotypic variance due to individual animal (between-animal) variation, known as repeatability of the trait, is also shown (Tables 3 and 4). Of the immunological traits, statistically significant repeatability estimates were found for NAb, monocytes, eosinophils, and % PBMC that were CD3, CD4, CD8, CD14, CD21, CD335 and γδ TCR positive (Table 3). For these traits, variation among animals accounted for 15 to 76% of the total phenotypic variance. Of the lactation traits, statistically significant (P<0.05) repeatability estimates were found for milk yield, fat yield, protein yield, feed intake, dry matter intake, live weight, empty body weight, body condition score and somatic cell count (Table 4). For these traits, variation among animals accounted for 15 to 82% of the total phenotypic variance. Because of this marked variation, lactation traits are routinely considered in animal selection and genetic improvement schemes. The fact that several immune traits exhibited similar levels of variation (Table 3) suggests that they, too, may be amenable to selection. Health events including mastitis, reproductive problems and lameness events recorded over the sampling period are summarised in Table 6.

**Table 3:** Immune traits: descriptive statistics, estimates of variance due to the animal effect and proportion of the total phenotypic variance due to the animal effect (between animal repeatability estimates).

Trait	No. Records	Mean	Std. Deviation	CV^1^%	Animal Variance	Rep^2^
NAb3(OD@492_)_	863	1.14	0.63	55.43	0.05*	0.15*
TNF-α^4^ (pg/ml)	860	556.07	1229.98	221.19	0.25	0.00
Haptoglobin (µg/ml)	860	156.21	271.74	173.96	2,438.04	0.04
% PBMC^5^	232	53.63	10.62	19.80	21.79	0.22
% CD3^+6^	236	29.90	8.54	28.58	29.43*	0.49*
% CD4^+6^	226	12.43	3.51	28.26	3.08*	0.29*
% CD8^+6^	226	7.96	3.00	37.68	6.27*	0.69*
% CD14^+6^	230	18.34	7.25	39.54	0.00	0.00
% CD21^+6^	230	38.03	10.27	27.01	55.45*	0.51*
% CD335^+6^	228	3.07	1.56	50.83	0.94*	0.43*
% γδ TCR^+6^	236	6.60	3.31	50.14	4.73*	0.61*
CD4^+^ : CD8^+^ ratio	226	1.72	0.66	38.25	0.26*	0.56*
% Lymphocytes^7^	231	41.21	10.50	25.48	17.01	0.20*
% Monocytes^7^	231	10.68	2.47	23.12	1.31*	0.24*
% Neutrophils^7^	232	39.43	10.53	26.71	16.79	0.18
% Eosinophils^7^	232	4.73	3.45	73.02	12.38*	0.76*

1 Coefficient of Variation;

2 Between animal repeatability of the trait;

3 Natural Antibodies;

4 Tumour Necrosis Factor-α;

5 %Peripheral Blood Mononuclear Cells;

6% of PBMC that were CD3, CD4, CD8, CD14, CD21, CD335 and γδ TCR positive;

7% of total leukocytes that were lymphocytes, monocytes, neutrophils or eosinophils.

* Significantly greater than 0 estimates (P<0.05).

**Table 4:** Lactation traits: descriptive statistics, estimates of variance due to the animal effect and proportion of the total phenotypic variance due to the animal effect (between animal repeatability estimates); data were weekly averaged daily records.

Trait	No. Records	Mean	Std. Deviation	CV^1^%	Animal Variance	Rep^2^
Milk yield (kg)	10,202	30.69	9.49	30.91	35.08*	0.71*
Fat yield (kg)	9,180	1.17	0.42	35.95	0.05*	0.45*
Protein yield (kg)	9,180	1.01	0.34	33.60	0.03*	0.44*
Feed intake (kg)	5,731	43.42	11.43	26.33	37.44*	0.39*
Dry matter intake (kg)	5,731	17.43	4.98	28.54	5.54*	0.39*
Feed to milk ratio	5,722	1.44	0.63	43.70	0.01	0.10
Dry matter to milk ratio	5,722	0.57	0.25	42.88	0.01	0.09
Live weight (kg)	9,716	590.61	83.01	14.05	2,529.29*	0.82*
Empty body weight (kg)	5,665	489.86	75.53	15.42	2,113.66*	0.76*
Body condition score (1–5)	8,377	2.08	0.43	20.80	0.09*	0.57*
Somatic cell count (1000/ml)	9,344	91.57	288.89	315.49	50,378.91*	0.58*

1 Coefficient of Variation;

2 Between animal repeatability of the trait;

* Significantly greater than 0 estimates (P<0.05).

**Table 5:** Reproductive performance traits: descriptive statistics.

Trait	No. Records	Mean	Std. Deviation	CV^1^%
Calving interval (d)	200	403.34	70.07	17.37
Days to first service (d)	240	69.32	27.47	39.62
Days to first heat (d)	240	65.34	30.41	46.54
Days first-last service (d)	240	70.17	82.99	118.27
Days first-second service (d)	219	25.10	25.42	101.26
Number of services	240	3.24	2.49	76.76
Dystocia (0/1)	240	0.35	0.48	
Stillbirth Rate (0/1)	240	0.02	0.14	

1 Coefficient of Variation.

**Table 6:** Health event traits: descriptive statistics.

Condition	Frequency	Mean	Std. Deviation	maximum
Clinical mastitis	0.01	0.29	0.58	3
Reproductive problems	0.04	0.87	0.89	4
Lameness	0.08	1.10	1.22	8

Frequency refers to the proportion of cows with a condition on the week of the immunological analysis; mean, standard deviation and maximum refer to number of distinct episodes per lactation.

### Effect of Genetic and Diet Group on Immune Traits

**Figure 1:**
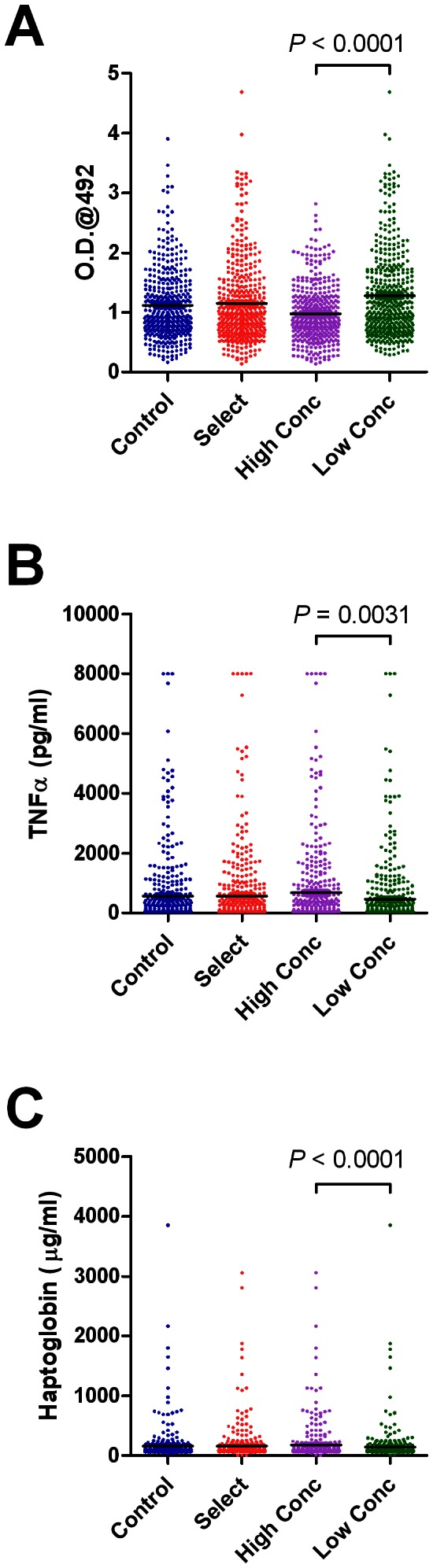
Effect of genetic and diet group on serological immune traits. Levels of natural antibodies (A), TNFα (B) and haptoglobin (C) in the serum of cows from control or select genetic groups and high concentrate (High Conc.) or low concentrate (Low Conc) diet groups. Data represents values recorded over the whole 8 month study period.

**Figure 2:**
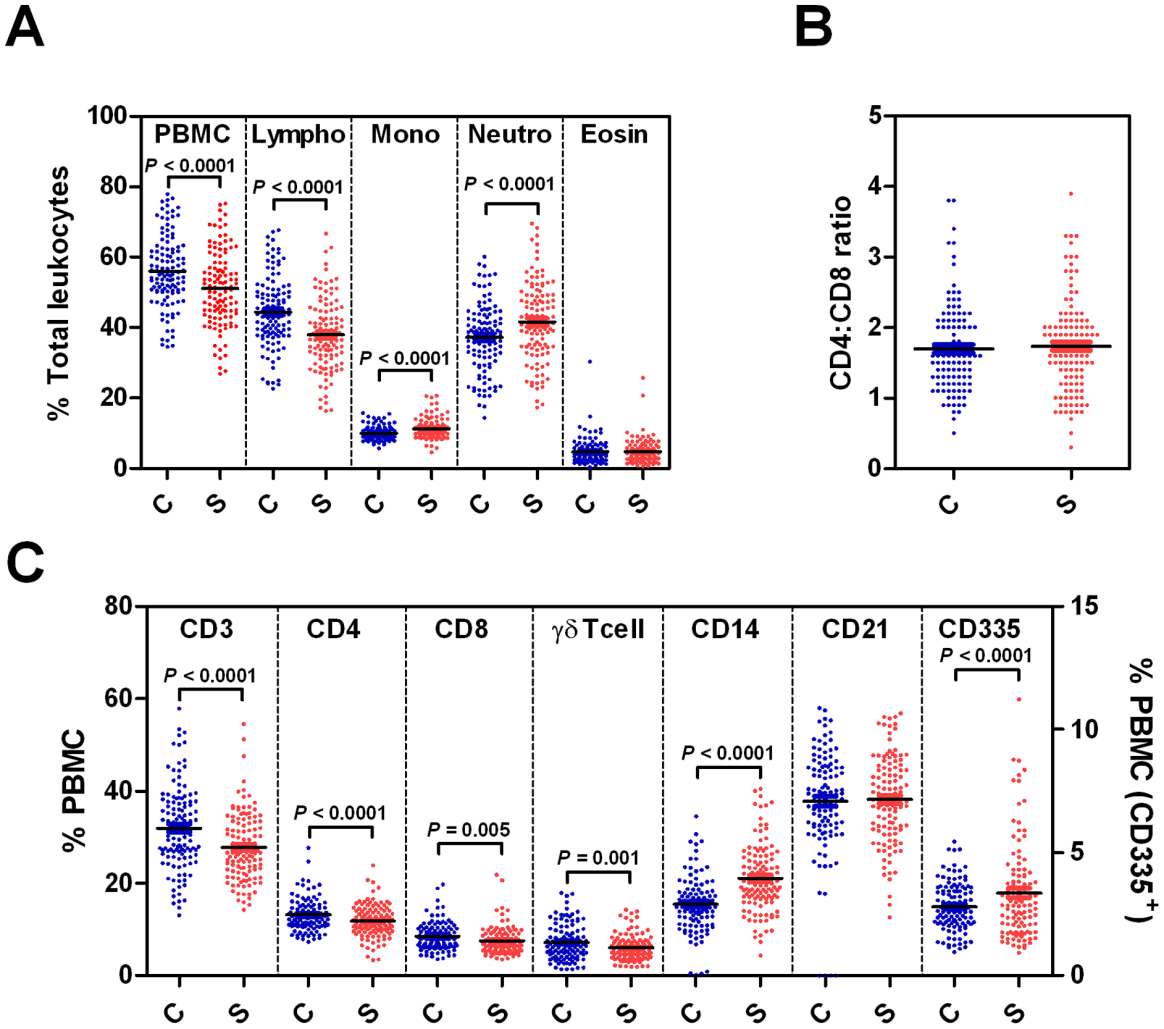
Effect genetic group on cellular immune traits. Differential leukocyte counts (A), CD4:CD8 ratios (B) and PBMC leukocyte subpopulations (C) in cows from either control (C) or select (S) genetic groups. Data represents values recorded over the whole 8 month study period.

The impact of genetic and diet group on the immune traits is shown in Figures 1 and 2. These effects were adjusted for all other sources of systematic variation included in the statistical models of analysis. The percentages of the various leukocyte subpopulations were only available for cows that belonged in the high concentrate diet group; therefore, no diet group effect was estimated in these cases. High concentrate rations represent the prevailing practice for intensively raised dairy cattle in countries with advanced livestock sectors. Therefore, results pertaining to immune traits derived with flow cytometry that are presented here may be extended to a wide range of the commercial dairy cattle population worldwide. A different set of outcomes may be expected in low-input livestock farming systems (e.g. organic) as far as these particular immune traits are concerned.

The effects of genetic and diet group on immune traits within serum are shown in Figure 1. Diet group had a significant (P<0.05) effect on the serum concentrations of NAb (Fig. 1A), TNF-α (Fig. 1B) and haptoglobin (Fig. 1C). Higher levels of concentrates fed were associated with decreased NAb levels but increased serum concentration of TNF-α and haptoglobin. Genetic group had no effect on any of these serum traits.

The effects of genetic group on blood leukocyte populations are shown in Figure 2. Genetic group had a significant effect on all leukocyte measurements except for the CD4^+^:CD8^+^ T cell ratio (Figure 2B), the percentage of CD21^+^ B cells within the PBMC population (Figure 2C) and the percentage of eosinophils within the total blood leukocyte population (Figure 2A). The select group of cows were associated with lower percentages of lymphocytes but higher percentages of monocytes and neutrophils within the total leukocyte population (Figure 2A). The select group of cows were also associated with lower % CD3^+^, CD4^+^ CD8^+^ and γδ TcR^+^ T lymphocytes but higher % CD14^+^ and CD335^+^ cells within the PBMC population (Figure 2C).

### Effect of Genetic and Diet Group on Health Event, Lactation and Reproductive Performance Traits

Analysis of the contribution of genetic background and dietary regime to health, lactation and reproductive performance was analysed by a repeatability mixed model. Results showed that cows in the select group had more distinct lameness episodes on average per lactation than cows in the control group. They also produced significantly (P<0.05) more daily milk, fat and protein; were heavier; had a lower body condition score; and consumed more feed and dry matter; but required less feed and dry matter to produce 1 kg of milk than control cows. Select group cows had significantly longer intervals between consecutive calvings and services; and between calving, first heat and service; and required more services per conception. There were no significant differences in milk somatic cell count, calving ease, stillbirth rate, and number of clinical mastitis and reproductive problem episodes between the two genetic groups.

Cows fed a high concentrate diet experienced more clinical mastitis and lameness episodes than cows in the low concentrate group. The high concentrate cows also produced significantly more daily milk, fat and protein; were heavier; had higher body condition score; and consumed more feed and dry matter. No differences in milk somatic cell count and reproductive performance were detected between the two diet groups.

### Correlations between Cellular and Serological Immune Traits

Potential relationships between the immunological measures made were assessed by a series of bivariate analyses within the repeatability model used for the overall analysis. These correlations were then subject to a strict Bonferroni correction to account for multiple testing. Only those correlations that remained significant (P<0.05) after Bonferroni correction are presented here. Table 7 summarises all significant (P<0.05) correlations between immunological traits. The significance of these correlations was confirmed by a series of 3-variate analyses including at least one PBMC percentage trait. Levels of circulating NAb were found to be negatively correlated with % PBMC and % lymphocytes, and positively correlated with the % CD14^+^ cells. Thus, animals with higher levels of NAb had lower % PBMC and lymphocytes but higher % CD14^+^ monocytes.

**Table 7:** Statistically significant (*P*<0.05) post Bonferroni correction phenotypic correlations among serological and cellular immune traits.

Immune trait 1	Immune trait 2	Phenotypic correlation	Standard error
NAb _(OD@492)1_	% PBMC^2^	−0.291	0.088
NAb _(OD@492)1_	% Lymphocytes^2^	−0.290	0.088
NAb _(OD@492)1_	% CD14^+3^	0.311	0.087

1 Natural Antibodies;

2 % of total leukocytes that were PBMC or lymphocytes;

3 % of PBMC that were CD14 positive.

### Correlations between Immune and Health Event Traits

In order to use the health event data for correlation analyses, health event traits were expressed as either 0 or 1 for absence or presence of the condition, respectively, on the specific week of measurement or were expressed as number of distinct episodes within a lactation period. Statistically significant (P<0.05) phenotypic correlations between immune profile and health event traits are shown in Table 8. Significant positive correlations were identified between clinical mastitis incidence on the week of sampling and levels of serum haptoglobin, and between the % CD335^+^ cells within the PBMC population and the number of lameness episodes within a lactation period. Statistically significant correlations that did not remain significant after the Bonferroni correction are summarised in Tables S1 and S2. Of the estimates in Table S2, of interest is the positive correlation between the number of clinical mastitis episodes in a lactation period and levels of serum haptoglobin, further supporting a link between haptoglobin and clinical mastitis.

**Table 8:** Statistically significant (*P*<0.05) post Bonferroni correction animal and phenotypic correlations between immune, health event, reproductive performance and lactation traits.

Immune profile trait^1^	Health event/Reproductive/Lactation trait	Phenotypic/animal correlation	Standard error
Haptoglobin (µg/ml)	Clinical mastitis^1^	0.324^a^	0.032
% CD335^+3^	Lameness episodes^2^	0.479^a^	0.113
% CD8^+3^	Calving interval	0.477	0.143
% PBMC^4^	Dystocia	−0.451^b^	0.123
% Lymphocytes^4^	Dystocia	−0.423^b^	0.126
CD4^+^ : CD8^+^ ratio^3^	Somatic cell count	−0.557^c^	0.158
CD4^+^ : CD8^+^ ratio^3^	Somatic cell count	−0.437^d^	0.134
NAb _(OD@492)5_	Feed intake	−0.215^e^	0.040
NAb _(OD@492)5_	Dry matter intake	−0.170^e^	0.041
% CD3^+3^	Feed to milk ratio	0.376^e^	0.093
% CD14^+3^	Feed intake	−0.405^e^	0.084
% γδ TCR^+^	Feed intake	0.401^e^	0.083
% Lymphocytes^4^	Feed intake	0.400^e^	0.084
NAb _(OD@492)5_	Dry matter intake	−0.130^f^	0.037
NAb _(OD@492)5_	Live weight	−0.119^f^	0.034

1 expressed as 0/1 on the week of the immune analysis;

2 expressed as number of distinct episodes in a lactation;

3 % of PBMC that were CD335, CD8, CD4, CD3, CD14 or γδ TCR positive;

4 % of total leukocytes that were PBMC or lymphocytes;

5 Natural Antibodies.

a phenotypic correlations between immune and health events traits;

b phenotypic correlations between immune and reproductive traits;

c animal correlations between immune and lactation traits measured on the same week; ^d^animal correlations between immune and lactation traits measured throughout lactation; ^e^phenotypic correlations between immune profile and lactation traits measured on the same week;

f phenotypic correlations between immune and lactation traits measured throughout lactation.

Results pertaining to number of clinical mastitis, reproductive problems and lameness episodes were based on the definition of distinct cases being 7, 21 and 28 days apart, respectively. A sensitivity analysis was conducted allowing these numbers to vary by as much as 20% on either direction. Parameter estimates from the additional analysis were always within the 95% confidence interval of the estimates shown in the Tables, thereby attesting to the robustness of the results.

### Correlations between Immune and Reproductive Performance Traits

Statistically significant (P<0.05) phenotypic correlations of immune traits with reproductive performance traits that remained significant after the Bonferroni correction are shown in Table 8. A positive correlation was found between % CD8^+^ cells within the PBMC population and calving interval, with higher % CD8^+^ associated with longer intervals between successive calvings. Dystocia, also an indication of welfare, was negatively associated with % PBMC and % lymphocytes within the blood leukocyte population. Statistically significant correlations of immune traits with reproduction that did not remain significant after the Bonferroni correction are summarised in Table S3.

### Correlations between Immune and Lactation Traits

Statistically significant (P<0.05) animal and phenotypic correlations between immune profile and lactation traits are shown in Table 8. Significant animal correlations were found between PBMC CD4^+^:CD8^+^ ratio and milk somatic cell count, both when the traits were measured on the same week or over the whole lactation period, with higher CD4^+^:CD8^+^ ratios associated with lower somatic cell counts. Significant phenotypic correlations were mainly related to feed consumption. Thus, higher amounts of feed or dry matter intake measured at the same time as the immunological analysis were associated with reduced NAb and % CD4^+^ within the PBMC population, but increased % γδ TCR^+^ cells and % lymphocytes. The feed intake to milk yield ratio measured at the same time as the immunological analysis was positively correlated with % CD3^+^ within the PBMC population, meaning that a higher percentage of T cells was associated with poorer conversion of feed to milk. Furthermore, increased dry matter intake and live weight throughout lactation were associated with reduced levels of serum NAb.

Several other correlations between immune and lactation traits were statistically significant but did not remain so after the Bonferroni correction. These estimates are summarised in the Supplementary Material. These include animal and phenotypic correlations between immune and lactation traits, measured on the same week of lactation (Tables S4 and S5, respectively), and measured throughout the entire lactation (Tables S6 and S7, respectively), for which the results are average weekly estimates across all weeks of lactation.

## Discussion

This study is the largest simultaneous and repeated evaluation of multiple immune traits in dairy cows in relation to health, fitness and performance, and provides a first insight into the utility of these immune measures for the improvement of animal health and consequent welfare. This study exploited a unique biological resource, the Langhill lines from the SRUC Crichton Dairy Research herd [12], [13], which represent the longest running large farmed animal selection experiment in the world. Uniquely, these cows are recorded from birth to death with routine and repeated collection of rigorously validated health and performance data (milk production, feed consumption, health events and reproductive traits) from individual animals in distinct genetic lines and diet groups. This allowed us to determine the association of immune traits with health events and production both at the experimental group level i.e. genetic and/or diet group, and at the individual animal level. It was also possible to estimate the between-animal repeatability for each trait and its correlation with health and fitness events at the time of sampling, thus providing an assessment of a particular trait’s potential for use in selective breeding programmes or as an immune biomarker of concurrent health events. Furthermore, similar analyses may also be informative in future studies of the epidemiology of infectious diseases in relation to animal genetics, productivity and husbandry.

At the group level, it was found that NAb levels were higher, while TNFα and haptoglobin levels were lower in the low concentrate/high forage diet group (Figure 1). NAb are an important antibody component of the innate immune response and are defined as antibodies which are spontaneously generated in animals without the need for antigenic stimulation. A high proportion of NAb bind to pathogen-associated molecular patterns present on microbial pathogens and it is thought that these antibodies represent a first line of defence by binding to pathogens once they have breached mucosal barriers [24], [25]. Recent studies in dairy cows support this hypothesis, with higher levels of NAb being associated with reduced incidence of mastitis [9]. As the low concentrate/high forage group had significantly fewer mastitis and lameness episodes, one interpretation might be that higher levels of NAb within this group are associated with an improved capacity of the innate immune system to respond to pathogenic challenge, with subsequently lower incidence of clinical/subclinical disease and consequently lower levels of TNFα and haptoglobin, both of which are associated with pro-inflammatory immune responses [15], [26]. The cause of increased NAb levels in the low concentrate/high forage diet group is unclear, although NAb levels in dairy cows have been previously shown to be influenced by diet with higher levels present in cows fed glucogenic compared to lipogenic diets [27]. Interestingly, genetic group had no significant effect on any serological immune traits.

This study also explored the effect of genetic group on cellular immune traits in cows fed on the high concentrate diet typical of the average UK dairy herd. Results show that the proportions of most leukocyte subsets were influenced by genetic group, with the select group having lower proportions of all lymphocyte classes but higher proportions of monocytes, neutrophils and CD335^+^ natural killer (NK) cells than the control group (Figure 2). The higher frequency of health events in the select group may suggest that suppression of lymphocyte numbers in these animals makes them more susceptible to disease, with a consequent increase in the proportion of inflammatory and innate immune effector cells (i.e. monocytes, neutrophils and NK cells) within the leukocyte population. Our results are consistent with human studies which demonstrate that peripheral blood lymphocyte levels are heritable and may influence disease progression [28], [29]. Alternatively, the higher frequency of health events within the select group may have resulted in increased levels of inflammatory/innate immune effector cells and the consequent reduction in the proportion of lymphocytes.

The group level results suggest that certain immune traits such as NAb or the proportions of circulating lymphocytes could be useful bio-markers for individual animals with increased resistance to disease, as these immune measures were higher in groups with lower frequencies of health events. However, when the data were analysed at the individual animal level, potential associations identified at the group level between immune traits and health events, particularly in relation to serological immune traits, were no longer apparent, even without the use of a Bonferroni correction which is rather strict and conservative. Furthermore, a number of the key associations seen at the individual animal level were not apparent when analysed at the group level. These results highlight the crucial importance of using individual animal health records when searching for immune traits associated with disease resistance.

At the individual animal level, the most significant correlation between immune and health traits was the negative association between CD4^+^:CD8^+^ ratio and SCC, a useful indicator of intra-mammary infection and mastitis [30], [31]. This association was significant, both at the phenotypic level and at the animal level, the latter indicating that the association between CD4^+^:CD8^+^ ratio and SCC was due to animal-specific effects including heritable factors. Furthermore, CD4^+^:CD8^+^ ratio exhibited a high level of between-animal variance and, more importantly, a high level of the proportion of total phenotypic variance was accounted for by the between-animal variance component. The latter is known as trait repeatability and encompasses genetic components, providing further evidence that this immune trait is potentially heritable in dairy cows. Studies in humans have estimated a heritability of the CD4^+^:CD8^+^ ratio of 65% [29] and have shown it to be associated with QTL within the major histocompatability complex [32]. A similar association between CD4^+^:CD8^+^ ratios and mastitis has been previously reported in a small scale study in Holstein dairy cows (n = 15) in which cows identified by previous clinical history and SCC as either resistant or susceptible to mastitis exhibited significantly different CD4^+^:CD8^+^ ratio profiles within both milk and blood [8]. This indicates that the CD4^+^:CD8^+^ ratio within circulating lymphocyte populations may be related to the CD4^+^:CD8^+^ ratio within mammary lymphocyte populations. How a greater CD4^+^:CD8^+^ ratio may aid in the protection against intra-mammary infections is unclear. One explanation is that CD4 helper T cells may be involved in protective immunity against intra-mammary pathogen challenge. This is supported by the observation that CD4 T cells are increased in the milk of cattle during naturally occurring or experimentally induced mastitis [33]–[35], and that interleukin (IL)-17, a recently discovered cytokine produced primarily by a subset of CD4^+^ cells, can induce expression of a number of immune defence genes by mammary epithelial cells [36]. Further evidence for a role of CD4^+^ in protection against mastitis is provided by the recent identification of a single-nucleotide polymorphism in the bovine CD4 gene, which is associated with SCC [37]. Another possibility is that CD8^+^ cells are associated with reduced immunity to intra-mammary infections. In milk obtained from either healthy cows or cows experimentally infected with *Staphylococcus aureus*, a population of CD8^+^ ACT2^+^ cells are present which are capable of suppressing CD4^+^ T cell responses [38] and it may be that lower CD4^+^:CD8^+^ ratios reflect increased levels of CD8^+^ACT2^+^ T cells within the mammary gland.

Analyses at the individual animal level also identified a significant positive correlation between the % CD335^+^ leukocytes within an individual animal and the number of lameness episodes during a lactation period. This correlation was significant at the phenotypic level, indicating that the correlation was a result of both genetic and environmental factors influencing both traits simultaneously, while the significant repeatability of % CD335^+^ (0.43) suggests that this trait is under some degree of genetic control. CD335, also known as NKp40, is exclusively expressed on the cell surface of resting and activated natural killer (NK) cells where it functions as an activating receptor [39], [40]. NK cells are important in innate defence against intracellular pathogens or tumours via spontaneous killing of infected or transformed cells [41]. Furthermore NK cells can modulate adaptive immune responses via early production of T helper type-1 (Th-1) associated cytokines or interactions with antigen presenting cells [42], [43]. How the proportion of NK cells is associated with lameness episodes is unclear. Lameness within this study was attributed to many diverse disease conditions, some associated with an infectious aetiology such as digital dermatitis (associated with *Dichelobacter nodosus* and possibly other infections agents [44]), and some with a largely non-infectious aetiology, such as slurry heel which is more associated with management practices [45]. Future analyses may further explore the relationship between % NK cells and different causes of lameness.

A number of significant correlations were identified at the phenotypic level between cellular traits and reproduction traits. These included a positive correlation between the % CD8^+^ T cell within the circulation and calving interval and a negative association between % PBMC and % lymphocytes and dystocia. CD8^+^ is a marker for cytotoxic T cells (CTLs) which are largely involved in the recognition and killing of cells infected with intracellular pathogens and are major effectors of Th-1 type responses [46]. There has been considerable interest in the human field in relation to the immunological basis of infertility, as successful pregnancy requires maternal immunological tolerance of a semi-allogeneic foetus. Studies in humans have demonstrated that a number of infertility problems such as recurrent pregnancy loss (RPL) and repeated implantation failure (RIF) are associated with Th-1 type responses and concomitant reductions in Th-2 type responses [47]–[50], presumably because Th-1 type responses are more damaging to the foetus. While no direct association has been found between % total CD8^+^ T cells and infertility in humans, the proportions of activated CD8^+^ T cells (as determined by co-expression of CD154) have been shown to be significantly higher in women with RPL and RIF than in fertile controls [51]. One possible explanation of the positive association between CD8^+^ T cells and calving interval is that higher % CD8^+^ T cells within individual cows reflected an increase in numbers of activated CD8^+^ T cells which could contribute to increased foetal death or implantation failure. The repeatability of % CD8^+^ was the highest among all the cellular traits measured in this study (0.69), suggesting that this may be a useful marker of cows with improved reproductive performance. The negative association between % PBMC and % lymphocytes and dystocia is less clear. It may be that higher % lymphocytes or % PBMC (the majority of which are lymphocytes) within the dam are associated with reduced birth-weights and therefore easier calvings, although there is currently little evidence to support this. Unlike % CD8^+^, these cellular traits exhibited either low within-animal repeatability (lymphocytes) or were not repeatable (PBMC), suggesting low levels of heritability. However, this low repeatability within trait combined with significant population correlation suggests that these immune bio-markers could reflect a temporal response to a particular immune system challenge.

Importantly, none of the cellular immune traits associated with improved health or reproductive performance (high CD4^+^:CD8^+^ ratio, low % of circulating CD8^+^ and CD335^+^ cells, high % PBMC and lymphocytes) were associated with reduced productivity such as poorer milk yield or reduced feed to milk conversion, suggesting that these markers could be used to select for animals with improved health without negative effects on productivity. Further work is required to provide accurate heritability estimates of these cellular immune traits to further explore their utility as selection markers for improved dairy cow health.

In addition to identifying a number of repeatable cellular immune traits associated with health and reproduction traits, this study also identified a significant positive correlation between clinical mastitis at the time of sampling and circulating levels of the acute phase protein haptoglobin, an immune trait with no significant trait repeatability. Although the low between-animal variance and repeatability for haptoglobin found in the present study renders it an unlikely biomarker for genetic selection, its significant association with mastitis identifies it as a potentially useful predictor of disease at the phenotypic level. Haptoglobin is a major acute phase protein in cattle which is synthesised primarily by the liver but also in mammary tissue [52], [53] in response to pro-inflammatory signals such as the cytokines IL-1β, IL-6 and TNF-α [15], [26], [54]–[56]. Levels of haptoglobin in both serum and milk have been shown to be increased in dairy cows with both experimentally-induced [52], [57], [58] or naturally-occurring cases of mastitis [59]–[61], increasing rapidly (within 1–2 days) following experimental intra-mammary infection with mastitis-causing pathogens. The results of this study are therefore consistent with previous research and lend further support for the use of haptoglobin as a potential biomarker of bovine mastitis.

Finally, this study allowed us to explore associations between immune traits and production traits related to milk production and nutrition, such as feed intake, body condition score, live-weight and feed to milk ratio (i.e. milk production efficiency). The most consistent associations identified were with circulating NAb levels which were negatively associated with a number of nutritional parameters including feed intake, dry matter intake (DMI), and live-weight throughout lactation. NAb levels have previously been related to dairy cow nutrition, being influenced by the type of diet fed and nutritional status, as defined by energy balance, with cows in negative energy balance having lower levels of NAb [9], [27]. While energy balance was not measured in this study, our findings suggest that poorer nutritional status as defined by feed intake and live-weight resulted in higher rather than lower levels of NAbs.

We showed significant associations between cellular immune traits and feed intake at the time of sampling, including a positive association between both % lymphocytes and % γδ TCR^+^ cells, and a negative association with % CD14^+^ monocytes. Furthermore, a positive relationship was identified between % cells expressing CD3, a pan-T cell marker, and feed to milk ratio at the time of sampling indicating that % circulating T cells are negatively associated with milk production efficiency. How these cellular traits are associated with feed intake or milk production efficiency is unclear, although it is possible that higher levels of monocytes and T cells at the time of sampling may be indicative of a concurrent immune response which may result in depressed feed intake or milk production efficiency.

It was apparent from this study that most associations between immune traits and health events involved cellular measures. As flow cytometry requires the preparation of live cells within a fixed time period prior to analysis, it is less practical than a serologically based test and we therefore explored correlations between serological and cellular immune traits. Unfortunately none of the cellular markers associated with improved health were strongly correlated with serum markers in this study and could therefore not be used as proxy markers for cellular traits. However, with increasing use of flow cytometry in clinical settings using high-throughput and robust methods [62], [63] it is now feasible to perform such measures reproducibly on a large scale and at minimal cost. Furthermore, as both serological and cellular immune traits become amenable to high throughput and low cost measurement, immune traits themselves may be useful as target phenotypes for genetic association studies aimed at discovering polymorphisms associated with improved health traits, where the correlated health trait is complex or difficult to record.

### Conclusions

To the best of our knowledge, this is the largest study to date reporting correlations of immune fitness with a wide range of health and production traits in dairy cows. While the results of this study need to be verified with larger, independent datasets they nevertheless provide a useful first insight into the utility of immunological measurements for the improvement of health, fitness and other important functions in dairy cattle. While the exact causal relationship between immune and health traits remains to be determined, these results clearly demonstrate that specific immune traits are related to dairy cow health and that these traits may be useful as markers for use in selective breeding programmes or genomic association studies aimed at improving health, or as phenotypic proxies for the on-farm management of animal health and fertility. In all cases, these results can directly impact on animal health and welfare, which have emerged as key public concerns that affect the confidence and attitude of consumers of animal products.

## Supporting Information

Table S1 Statistically significant (P<0.05) phenotypic correlations between immune and health event traits expressed as 0/1 on the week of the immune analysis, that did not remain significant after the Bonferroni correction.
(DOCX)

Table S2 Statistically significant (P<0.05) phenotypic correlations between immune and health traits expressed as number of distinct episodes in a lactation, that did not remain significant after the Bonferroni correction.
(DOCX)

Table S3 Statistically significant (P<0.05) phenotypic correlations between immune and reproductive traits, that did not remain significant after the Bonferroni correction.
(DOCX)

Table S4 Statistically significant (P<0.05) animal correlations between immune and lactation traits measured on the same week, that did not remain significant after the Bonferroni correction.
(DOCX)

Table S5 Statistically significant (P<0.05) phenotypic correlations between immune and lactation traits measured on the same week, that did not remain significant after the Bonferroni correction.
(DOCX)

Table S6 Statistically significant (P<0.05) animal correlations between immune and lactation traits measured throughout the lactation, that did not remain significant after the Bonferroni correction.
(DOCX)

Table S7 Statistically significant (P<0.05) phenotypic correlations between immune and lactation traits throughout the lactation, that did not remain significant after the Bonferroni correction.
(DOCX)

Appendix S1 Statistical models of analysis.
(DOCX)
