# Supplementary material for: Identification of Immune Traits Correlated with Dairy Cow Health, Reproduction and Productivity
Source: PLoS One. 2013 Jun 12;8(6):e65766. doi: 10.1371/journal.pone.0065766 (PMC3680463; doi:10.1371/journal.pone.0065766)
Supplement: Table S1 — Statistically significant (P<0.05) phenotypic correlations between immune and health event traits expressed as 0/1 on the week of the immune analysis, that did not remain significant after the Bonferroni correction. (DOCX) [file pone.0065766.s001.docx]

| **Table S1.** Statistically significant (P<0.05) phenotypic correlations between immune and health event traits expressed as 0/1 on the week of the immune analysis, that did not remain significant after the Bonferroni correction. | | | |
| --- | --- | --- | --- |
| Immune trait | Health event trait | Phenotypic correlation | Standard error |
| NAb _(OD@492)_ | Clinical mastitis | -0.070 | 0.035 |
| % CD4^+1^ | Reproductive problems | 0.186 | 0.085 |
| CD4^+^ : CD8^+^ ratio | Lameness | 0.233 | 0.082 |
| NAb = natural antibodies; ^1^ % of PBMC that are CD4 positive. | | | |
