# Supplementary material for: Identification of Immune Traits Correlated with Dairy Cow Health, Reproduction and Productivity
Source: PLoS One. 2013 Jun 12;8(6):e65766. doi: 10.1371/journal.pone.0065766 (PMC3680463; doi:10.1371/journal.pone.0065766)
Supplement: Table S2 — Statistically significant (P<0.05) phenotypic correlations between immune and health traits expressed as number of distinct episodes in a lactation, that did not remain significant after the Bonferroni correction. (DOCX) [file pone.0065766.s002.docx]

| **Table S2.** Statistically significant (P<0.05) phenotypic correlations between immune and health traits expressed as number of distinct episodes in a lactation, that did not remain significant after the Bonferroni correction. | | | |
| --- | --- | --- | --- |
| Immune trait | Health event trait | Phenotypic correlation | Standard error |
| Haptoglobin (μg/ml) | Clinical mastitis episodes | 0.191 | 0.064 |
| % CD8^+1^ | Clinical mastitis episodes | 0.212 | 0.074 |
| % CD8^+1^ | Reproductive episodes | 0.422 | 0.174 |
| % CD14^+1^ | Reproductive episodes | -0.372 | 0.162 |
| % γδ TCR^+1^ | Reproductive episodes | 0.356 | 0.164 |
| % Monocytes^2^ | Reproductive episodes | -0.426 | 0.157 |
| % Eosinophils^2^ | Reproductive episodes | 0.455 | 0.160 |
| ^1^ % of PBMC that are CD8, CD14 or γδ TCR positive; ^2^ % of total leukocytes that are lymphocytes. | | | |
