# Supplementary material for: Identification of Immune Traits Correlated with Dairy Cow Health, Reproduction and Productivity
Source: PLoS One. 2013 Jun 12;8(6):e65766. doi: 10.1371/journal.pone.0065766 (PMC3680463; doi:10.1371/journal.pone.0065766)
Supplement: Table S3 — Statistically significant (P<0.05) phenotypic correlations between immune and reproductive traits, that did not remain significant after the Bonferroni correction. (DOCX) [file pone.0065766.s003.docx]

| **Table S3.** Statistically significant (P<0.05) phenotypic correlations between immune and reproductive traits, that did not remain significant after the Bonferroni correction. | | | |
| --- | --- | --- | --- |
| Immune trait^1\^ | Reproductive trait | Phenotypic correlation | Standard error |
| Haptoglobin (μg/ml) | Days first-second service | 0.153 | 0.062 |
| % PBMC^1^ | Calving interval | 0.321 | 0.163 |
| % PBMC^1^ | Days to first service | 0.290 | 0.136 |
| % CD3^+2^ | Calving interval | 0.402 | 0.147 |
| % CD4^+2^ | Calving interval | 0.445 | 0.151 |
| % Lymphocytes^1^ | Days to first service | 0.273 | 0.135 |
| % Monocytes^1^ | Number of services | 0.312 | 0.142 |
| % Neutrophils^1^ | Days to first service | -0.277 | 0.136 |
| % Neutrophils^1^ | Dystocia | 0.407 | 0.129 |
| % Eosinophils^1^ | Stillbirth rate | 0.398 | 0.143 |
| ^1^ % of total leukocytes that are PBMC, lymphocytes, monocytes, neutrophils or eosinophils; ^2^ % of PBMC that are CD3 or CD4 positive. | | | |
