# Supplementary material for: Identification of Immune Traits Correlated with Dairy Cow Health, Reproduction and Productivity
Source: PLoS One. 2013 Jun 12;8(6):e65766. doi: 10.1371/journal.pone.0065766 (PMC3680463; doi:10.1371/journal.pone.0065766)
Supplement: Table S4 — Statistically significant (P<0.05) animal correlations between immune and lactation traits measured on the same week, that did not remain significant after the Bonferroni correction. (DOCX) [file pone.0065766.s004.docx]

| **Table S4.** Statistically significant (P<0.05) animal correlations between immune and lactation traits measured on the same week, that did not remain significant after the Bonferroni correction. | | | |  |
| --- | --- | --- | --- | --- |
| Immune trait | Lactation trait | Animal correlation | Standard error |  |
| % PBMC^1^ | Body condition score | -0.569 | 0.287 |  |
| % CD3^+2^ | Protein yield | -0.536 | 0.238 |  |
| % CD3^+2^ | Dry matter intake | -0.699 | 0.340 |  |
| % γδ TCR^+2^ | Protein yield | -0.576 | 0.208 |  |
| % Monocytes^1^ | Somatic cell count | 0.538 | 0.270 |  |
| % Eosinophils^1^ | Feed intake | -0.577 | 0.222 |  |
| % Eosinophils^1^ | Dry matter intake | -0.530 | 0.256 |  |
| ^1^ % of total leukocytes that are PBMC, monocytes or eosinophils; ^2^ % of PBMC that are CD3 or γδ TCR positive. | | | | |
