# Supplementary material for: Identification of Immune Traits Correlated with Dairy Cow Health, Reproduction and Productivity
Source: PLoS One. 2013 Jun 12;8(6):e65766. doi: 10.1371/journal.pone.0065766 (PMC3680463; doi:10.1371/journal.pone.0065766)
Supplement: Table S5 — Statistically significant (P<0.05) phenotypic correlations between immune and lactation traits measured on the same week, that did not remain significant after the Bonferroni correction. (DOCX) [file pone.0065766.s005.docx]

| **Table S5.** Statistically significant (P<0.05) phenotypic correlations between immune and lactation traits measured on the same week, that did not remain significant after the Bonferroni correction. | | | |
| --- | --- | --- | --- |
| Immune trait | Lactation trait | Phenotypic correlation | Standard error |
| NAb _(OD@492)_ | Fat yield | -0.101 | 0.037 |
| NAb _(OD@492)_ | Protein yield | -0.102 | 0.037 |
| NAb _(OD@492)_ | Feed to milk ratio | -0.118 | 0.043 |
| NAb _(OD@492)_ | Dry matter to milk ratio | -0.093 | 0.043 |
| Haptoglobin (μg/ml) | Dry matter intake | -0.081 | 0.040 |
| Haptoglobin (μg/ml) | Somatic cell count | 0.101 | 0.039 |
| % PBMC^1^ | Somatic cell count | -0.173 | 0.088 |
| % CD3^+2^ | Protein yield | -0.183 | 0.083 |
| % CD3^+2^ | Dry matter to milk ratio | 0.250 | 0.101 |
| % CD4^+2^ | Feed to milk ratio | 0.223 | 0.111 |
| % CD4^+2^ | Dry matter to milk ratio | 0.219 | 0.107 |
| % CD8^+2^ | Somatic cell count | 0.188 | 0.090 |
| CD4^+^ to CD8^+^ ratio | Somatic cell count | -0.235 | 0.089 |
| % CD14^+2^ | Dry matter intake | -0.247 | 0.097 |
| % CD14^+2^ | Feed to milk ratio | -0.249 | 0.104 |
| % CD21^+2^ | Feed to milk ratio | -0.313 | 0.096 |
| % CD21^+2^ | Dry matter to milk ratio | -0.254 | 0.097 |
| % CD21^+2^ | Body condition score | -0.217 | 0.078 |
| % γδ TCR^+2^ | Dry matter intake | 0.312 | 0.092 |
| % γδ TCR^+2^ | Feed to milk ratio | 0.216 | 0.107 |
| % γδ TCR^+2^ | Empty body weight | -0.210 | 0.088 |
| % γδ TCR^+2^ | Body condition score | 0.200 | 0.077 |
| % Lymphocytes^1^ | Dry matter intake | 0.209 | 0.099 |
| % Lymphocytes^1^ | Body condition score | -0.158 | 0.080 |
| % Lymphocytes^1^ | Somatic cell count | -0.186 | 0.088 |
| % Neutrophils^1^ | Feed intake | -0.309 | 0.092 |
| % Eosinophils^1^ | Milk yield | -0.158 | 0.078 |
| % Eosinophils^1^ | Fat yield | -0.180 | 0.084 |
| % Eosinophils^1^ | Protein yield | -0.190 | 0.083 |
| % Eosinophils^1^ | Feed intake | -0.207 | 0.097 |
| % Eosinophils^1^ | Dry matter intake | -0.203 | 0.098 |
| % Eosinophils^1^ | Body condition score | 0.152 | 0.078 |

^1^ % of total leukocytes that are PBMC, lymphocytes, neutrophils or eosinophils; ^2^ % of PBMC that are CD3, CD4, CD8, CD14, CD21 or γδ TCR positive.
