# Supplementary material for: Identification of Immune Traits Correlated with Dairy Cow Health, Reproduction and Productivity
Source: PLoS One. 2013 Jun 12;8(6):e65766. doi: 10.1371/journal.pone.0065766 (PMC3680463; doi:10.1371/journal.pone.0065766)
Supplement: Table S6 — Statistically significant (P<0.05) animal correlations between immune and lactation traits measured throughout the lactation, that did not remain significant after the Bonferroni correction. (DOCX) [file pone.0065766.s006.docx]

| **Table S6.** Statistically significant (P<0.05) animal correlations between immune and lactation traits measured throughout the lactation, that did not remain significant after the Bonferroni correction. | | | |
| --- | --- | --- | --- |
| Immune trait | Lactation trait | Animal correlation | Standard error |
| % CD8^+1^ | Somatic cell count | 0.280 | 0.144 |
| % CD14^+1^ | Live weight | -0.477 | 0.180 |
| % CD14^+1^ | Empty body weight | -0.525 | 0.167 |
| % γδ TCR^+1^ | Protein yield | -0.277 | 0.131 |
| % Monocytes^2^ | Live weight | -0.321 | 0.154 |
| % Monocytes | Empty body weight | -0.349 | 0.159 |
| ^1^ % of PBMC that are CD8, CD14 or γδ TCR positive. ^2^ % of total leukocytes that are monocytes | | | |
