# Supplementary material for: Identification of Immune Traits Correlated with Dairy Cow Health, Reproduction and Productivity
Source: PLoS One. 2013 Jun 12;8(6):e65766. doi: 10.1371/journal.pone.0065766 (PMC3680463; doi:10.1371/journal.pone.0065766)
Supplement: Table S7 — Statistically significant (P<0.05) phenotypic correlations between immune and lactation traits throughout the lactation, that did not remain significant after the Bonferroni correction. (DOCX) [file pone.0065766.s007.docx]

| **Table S7.** Statistically significant (P<0.05) phenotypic correlations between immune and lactation traits throughout the lactation, that did not remain significant after the Bonferroni correction. | | | |
| --- | --- | --- | --- |
| Immune trait | Lactation trait | Phenotypic correlation | Standard error |
| NAb _(OD@492)_ | Feed intake | -0.115 | 0.038 |
| NAb _(OD@492)_ | Dry matter to milk ratio | -0.083 | 0.040 |
| Haptoglobin (μg/ml) | Feed intake | -0.104 | 0.038 |
| Haptoglobin (μg/ml) | Dry matter intake | -0.086 | 0.036 |
| Haptoglobin (μg/ml) | Empty body weight | 0.084 | 0.036 |
| Haptoglobin (μg/ml) | Somatic cell count | 0.108 | 0.036 |
| % PBMC^1^ | Somatic cell count | -0.170 | 0.070 |
| CD4^+^ : CD8^+^ ratio | Feed intake | -0.212 | 0.091 |
| CD4^+^ : CD8^+^ ratio | Dry matter intake | -0.188 | 0.089 |
| CD4^+^ : CD8^+^ ratio | Somatic cell count | -0.200 | 0.071 |
| % CD14^+2^ | Live weight | -0.170 | 0.069 |
| % CD14^+2^ | Empty body weight | -0.151 | 0.072 |
| % CD21^+2^ | Body condition score | -0.161 | 0.069 |
| % CD21^+2^ | Somatic cell count | -0.153 | 0.071 |
| % γδ TCR^+2^ | Feed intake to milk ratio | -0.237 | 0.091 |
| % γδ TCR^+2^ | Dry matter to milk ratio | -0.208 | 0.090 |
| % γδ TCR^+2^ | Body condition score | 0.146 | 0.068 |
| % Lymphocytes^1^ | Somatic cell count | -0.215 | 0.069 |
| % Neutrophils^1^ | Somatic cell count | 0.144 | 0.071 |
| % Eosinophils^1^ | Fat yield | -0.195 | 0.070 |
| % Eosinophils^1^ | Protein yield | -0.138 | 0.069 |
| ^1^ % of total leukocytes that are PBMC, lymphocytes, neutrophils or eosinophils; ^2^ % of PBMC that are CD14, CD21 or γδ TCR positive. | | | |
