# Supplementary material for: Identification of Immune Traits Correlated with Dairy Cow Health, Reproduction and Productivity
Source: PLoS One. 2013 Jun 12;8(6):e65766. doi: 10.1371/journal.pone.0065766 (PMC3680463; doi:10.1371/journal.pone.0065766)
Supplement: Appendix S1 — Statistical models of analysis. (DOCX) [file pone.0065766.s008.docx]

**Appendix S1.** Statistical models of analysis

1. Repeatability mixed linear model for the analysis of immune traits, and weekly health and lactation traits corresponding to the week of the immune measurement:

$$Y_{ijklmno}=\mu+G_{i}+D_{j}+YM_{k}+LA_{l}+W_{m}+A_{n}+e_{ijklmno}$$

Y_ijklmno_ = Animal record

µ = Overall mean

G_i_ = Fixed effect of i^th^ genetic line (i=1, 2)

D_j_ = Fixed effect of j^th^ diet group (j=1, 2)

YM_k_ = Fixed effect of k^th^ year by month of calving interaction (k=1, 25)

LA_l_ = Fixed effect of l^th^ lactation number (l=1, 3) by age at calving interaction

W_m_ = Fixed effect of m^th^ week of lactation when the record was taken (m=1, 44)

A_n_ = Random effect of n^th^ individual cow (mean=0, variance=V_a_)

e_ijklmno_ = Random residual effect (mean=0, variance=V_e_)

1. Fixed linear model for the analysis of number of health episodes and reproductive traits:

$$Y_{ijklmo}=\mu+G_{i}+D_{j}+YM_{k}+LA_{l}+W_{m}+e_{ijklmo}$$

Y_ijklmo_ = Animal record

µ = Overall mean

G_i_ = Fixed effect of i^th^ genetic line (i=1, 2)

D_j_ = Fixed effect of j^th^ diet group (j=1, 2)

YM_k_ = Fixed effect of k^th^ year by month of calving interaction (k=1, 25)

LA_l_ = Fixed effect of l^th^ lactation number (l=1, 3) by age at calving interaction

W_m_ = Fixed effect of m^th^ week of lactation when the record was taken (m=1, 44)

e_ijklmo_ = Random residual effect (mean=0, variance=V_e_)

1. Random regression model for repeated measures analysis of lactation traits across the entire lactation:

$$Y_{ijkqlmnpo}=\mu+G_{i}+D_{j}+YM_{k}+YMR_{q}+LA_{l}+\sum_{p=0}^{2} b_{p}P_{p}W_{m}+\sum_{p=0}^{2} A_{pn}P_{p}W_{m}+e_{ijkqlmnpo}$$

Y_ijkqlmnpo_ = Animal record

µ = Overall mean

G_i_ = Fixed effect of i^th^ genetic line (i=1, 2)

D_j_ = Fixed effect of j^th^ diet group (j=1, 2)

YM_k_ = Fixed effect of k^th^ year by month of calving interaction (k=1, 25)

YMRq = Fixed effect of q^th^ year by month of record interaction (q=1, 28)

LA_l_ = Fixed effect of l^th^ lactation number (l=1, 3) by age at calving interaction

W_m_ = Fixed effect of m^th^ week of lactation when the record was taken (m=1, 44)

b_p_ = Fixed regression coefficient on week of lactation

P_p_ = p^th^ orthogonal polynomial of week of lactation (p=order of polynomial),

A_pn_ = Random regression coefficient on week of lactation associated with the random effect of the n^th^ individual cow

e_ijqklmnpo_ = Random residual effect (mean=0, variance=V_e_)
